# Heat-Moderating Effects of Bus Stop Shelters and Tree Shade on Public Transport Ridership

**DOI:** 10.3390/ijerph18020463

**Published:** 2021-01-08

**Authors:** Kevin Lanza, Casey P. Durand

**Affiliations:** 1 Michael and Susan Dell Center for Healthy Living, School of Public Health in Austin, The University of Texas Health Science Center at Houston, Austin, TX 78701, USA; 2 Michael and Susan Dell Center for Healthy Living, Department of Health Promotion & Behavioral Sciences, School of Public Health in Houston, The University of Texas Health Science Center at Houston, Houston, TX 77030, USA; Casey.P.Durand@uth.tmc.edu

**Keywords:** public transit, climate change adaptation, resilience, health equity, urban tree canopy, green infrastructure, built environment, temperature

## Abstract

Rising temperatures threaten the resilience of public transit systems. We determined whether bus stop shelters and tree canopy surrounding bus stops moderated the effect of warm season temperatures on ridership in Austin, Texas, and whether shelters and trees were equitably distributed. For bus stops (n = 2271) of Capital Metropolitan Transportation Authority, boardings per bus were measured 1 April–30 September 2019. Air temperature data originated from the Camp Mabry weather station. Tree canopy was calculated by classification of high-resolution aerial imagery from the National Agriculture Imagery Program. Data on race, ethnicity, poverty level, median age, and bus commuters within census tracts of bus stops originated from the 2014–2018 American Community Survey. Using multilevel negative binomial regression models, we found that shelters did not moderate the effect of high temperatures on ridership (*p* > 0.05). During high temperatures, each one-percent increase in tree canopy was associated with a lesser decrease (1.6%) in ridership compared to if there were no trees (1.7%) (*p* < 0.001). In general, shelters and trees were equitably distributed. Insignificant or modest effects of shelters and trees on ridership during high temperatures may be attributed to the transit dependency of riders. For climate change adaptation, we recommend tree planting at bus stops to protect from ridership losses and unhealthy exposure to extreme heat.

## 1. Introduction

Public transit has been established as superior to the automobile for its environmental and health benefits [1]. Compared to automobiles, public transit has been found to emit, on average, less than half the amount of greenhouse gases per passenger mile [2]. Along with climate change mitigation, shifting travel modes from automobiles to bus transit has been shown to reduce concentrations of harmful air pollutants, including carbon monoxide, nitrogen oxides, and particulate matter [3,4]. The per-mile crash rate of public transit is more than ten times lower than that of automobiles, and transit ridership has been found to be negatively associated with traffic fatalities [5]. Researchers have found public transportation and driving alone to exhibit negative and positive associations with mental health issues (*p* < 0.01), respectively [6]. Shifting from automobile use to public transit is also an opportunity for more physical activity: researchers have determined transit users to be 7.3 (95% CI: 2.6–20.1) times more likely to reach recommended levels of physical activity than non-transit users [7].

In the United States, more than two-thirds of public transit users have been found to walk to their stop or station [8]. Reaching the stop, station, or trip destination and waiting for the public transit vehicle to arrive is time potentially spent outdoors, exposed to prevailing weather conditions. Researchers have assessed the relations between weather and transit ridership, with mixed results based on weather condition (i.e., rain, snow, humidity, wind, and temperature), transit mode (i.e., bus, rail, and ferry), and weekdays versus weekends [9,10,11,12,13,14,15,16,17,18,19,20]. Specific to bus transit, the majority of studies have found rain, snow, and humidity to exhibit significant negative associations with ridership [10,13,14,19,20], with select studies finding no statistically significant associations on weekdays, suggesting weekday users are engaging in non-discretionary travel [15,16]. Researchers have found mixed associations between wind and bus ridership [10,14,15,16,19], with differences attributed to wind speed. Temperatures have been found to exhibit significant positive associations or insignificant associations with bus ridership [10,15,16]; however, temperatures at the low and high extremes have been found to exhibit significant negative associations with bus ridership [11,13,14,19,20]. For example, researchers found that bus ridership in Lane County, Oregon, decreased by 0.3% when daily maximum air temperatures reached or exceeded 29 °C, compared to milder temperatures (*p* < 0.05) [11]. Similarly, researchers found that daily boardings at unsheltered bus stops in Salt Lake City, Utah, decreased by 0.4% on days when temperatures averaged 23 °C or above (*p* < 0.01) [20].

High temperatures are a present and future challenge to the resilience of public transit systems. Globally, the last five years of the 1880–2019 historical climate record have been the warmest ever recorded [21], and the frequency, intensity, and duration of heat waves have increased over time [22]. Without additional climate change mitigation efforts, global temperatures are expected to increase 3.7–4.8 °C above pre-industrial levels by 2100 [23]. Furthermore, cities—home to more than half the world’s population [24]—experience higher temperatures than nearby rural areas due to the urban heat island effect [25]. This urban heat is inequitably distributed in the United States, with communities characterized by low levels of education and high levels of poverty and non-white populations disproportionately exposed to high temperatures [26]. These same US populations with high heat exposure are more likely to take public transportation than other populations [27].

With the threat of high temperatures on bus ridership, bus stop shelters—structures with roofs constructed at bus stops that provide protection from inclement weather conditions, such as rain and strong sun—are a potential climate change adaptation strategy. Researchers have found that bus stops with shelters have higher ridership during extreme low and high temperatures and during heavy precipitation than bus stops without shelters in Salt Lake City, Utah (*p* < 0.01) [20]. In addition, these researchers found that bus stop shelters were more likely to be installed in areas with higher household incomes and lower percentages of white residents (*p* < 0.01). Along with shelters, another potential climate change adaptation strategy for public transit systems is planting trees near bus stops. Tree planting is a proven heat management strategy [25,28], with researchers finding street trees to reduce direct and thermal radiation by 58% [29]. Yet, this valuable green infrastructure has been found to be inequitably distributed: in 25 US cities, researchers have measured less tree canopy in census tracts composed of majority low-income and Latinx populations than in other tracts [30].

To our knowledge, no previous studies have investigated (a) how bus stop shelters moderate the relation between temperature and ridership during the warm season in humid subtropical climates; (b) how trees surrounding bus stops moderate the relation between temperature and ridership; or (c) the spatial distribution of trees surrounding bus stops in relation to the sociodemographic characteristics of the surrounding community. As such, the main aims of this work were to determine whether bus stop shelters and tree canopy surrounding bus stops (a) ameliorated the effect of temperatures on bus ridership during the warm season in the city of Austin, Texas, and (b) were equitably distributed. In testing these aims, we found that temperatures exhibited a significant negative association with bus ridership, and tree canopy slightly moderated the effect of high temperatures on ridership. Both shelters and tree canopy were equitably distributed based on race, ethnicity, and poverty level of the population within tracts of bus stops.

## 2. Materials and Methods

### 2.1. Study Setting

For this study, we focused on the city of Austin, Texas, during the six-month warm season from 1 April 2019 through 30 September 2019. Located in the southern US, Austin spans approximately 771.6 km^2^, and was the eleventh most populous US city in 2019 with an estimated 978,908 inhabitants [31,32]. The city experiences a humid subtropical climate: long, hot summers and short, mild winters, with around 86.4 cm of rainfall annually [33,34]. On average, daily temperatures in Austin reach 32.4 °C from April through September and 36.1 °C in August, the warmest month [35].

**Figure 1:**
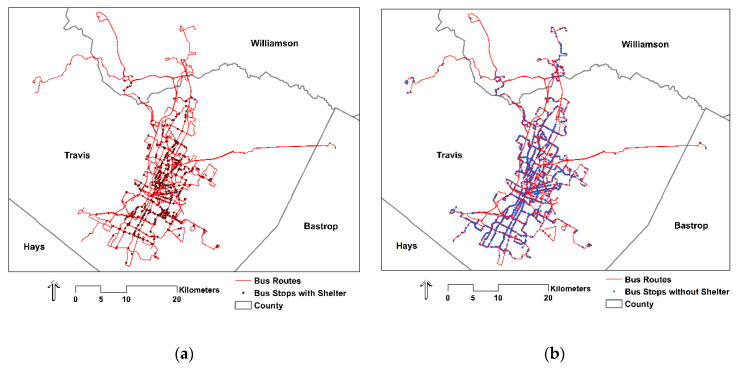
(**a**) Capital Metro bus stops with shelter (n = 661); (**b**) Capital Metro bus stops without shelter (n = 1610).

Capital Metropolitan Transit Authority (Capital Metro) is the regional public transportation provider for Austin. Established in 1985, Capital Metro serves approximately 1,300,518 people over a 1409.0 km^2^ service area with bus, rail, and paratransit [36]. In 2019, Capital Metro bus services constituted 95.3% of the 30.5 million total boardings across all services [37]. Specifically, Capital Metro has three primary bus services—MetroBus, MetroRapid, and MetroExpress—that utilize 83 bus routes and approximately 2300 bus stops, of which about one-third have shelters (Figure 1). Constituting 75.9% of all Capital Metro boardings in 2019 [37], MetroBus is the centerpiece and most extensive service of the Capital Metro system. MetroBus routes include a subset of 12 high-frequency routes with 10-min and 15-min headways on weekdays and weekends, respectively. Constituting 16.8% of all Capital Metro boardings in 2019 [37], MetroRapid is two additional high-frequency routes that travel north to south through central Austin. Lastly, MetroExpress is a commuter bus service connecting north Austin and outlying areas with central Austin, and constituted 2.6% of all Capital Metro boardings in 2019 [37].

### 2.2. Measurement of Bus Ridership

Our variable for bus ridership was the number of boardings per bus between 13:00 and 18:00 each study day (i.e., 1 April 2019 through 30 September 2019) for each bus stop. Ridership data originated from automatic passenger counters—sensors installed by Capital Metro above bus doors that record passenger boardings and alightings at each bus stop [38]. Boardings included those from MetroBus, MetroRapid, and MetroExpress, and bus stops can serve multiple routes. For each bus stop, we added the number of passengers that boarded through all doors each time a bus arrived between 13:00 and 18:00 each study day, and then divided this sum by the number of buses that arrived over that same time period. We selected 13:00–18:00 to best align with our measurement of temperature in the study.

### 2.3. Measurement of Temperature

Air temperature data originated from a weather station (30.3208°, −97.7604°; station ID: USW00013958) of the Global Historical Climatology Network installed at Camp Mabry, a military installation in central Austin. For the six-month study period, we downloaded the daily summaries dataset for this weather station from the National Oceanic and Atmospheric Administration’s National Centers for Environmental Information [35]. With no historical hourly data available, we used daily maximum temperature—rather than average or minimum temperature—because maximum temperatures occur during daytime when most travel occurs, and the high resolution of ridership data permitted us to focus on the time period with the highest temperatures, 13:00–18:00. Hourly normals for temperature (i.e., average air temperature for each hour of the year from 1981–2010) for April through September from the Camp Mabry weather station showed that diurnal temperatures peaked at 15:00 (i.e., mean = 31.1 °C; standard deviation = 3.3 °C), and were relatively stable between 13:00 (i.e., mean = 30.2 °C; standard deviation = 3.4 °C) and 18:00 (i.e., mean = 29.7 °C; standard deviation = 3.2 °C) [35]. We developed two variables for temperature: Tmax as a continuous variable of daily maximum air temperature (°C) and Hi-T Day as a binary variable for high-temperature days (i.e., 1 = high-temperature day; 0 = not high-temperature day). We defined high-temperature days as those where daily maximum air temperature met or exceeded the 90th percentile of daily maximum air temperatures (i.e., 36.3 °C) from normals of daily maximum air temperature for April through September from the Camp Mabry weather station [35], with the 90th percentile selected as the threshold for its use in previous studies [20,39].

### 2.4. Measurement of Bus Stop Shelters

The typical Capital Metro bus stop shelter consists of a metal roof, with dimensions of 3.0 m by 3.0 m; 2.1 m by 4.3 m; or 1.5 m by 3.7 m, over a metal bench, and is accompanied by a litter container [40]. Capital Metro shelters can have a variety of accessories, including electronic messaging signs with route and arrival information, security cameras, and lights. Capital Metro service guidelines and standards state that bus stops generating at least 50 daily boardings qualify for a shelter, and bus stops with at least 25 daily boardings may receive a shelter if also meeting at least three criteria: (a) adjacent to apartments with 250+ units; (b) adjacent to hospitals or social service agencies; (c) adjacent to major activity or employment centers; (d) adjacent to schools located at route intersections; and (e) service frequency greater than 30 min [41]. Capital Metro provided data on the number of shelters at each bus stop [40], which we transformed to a binary variable (i.e., 1 = presence of shelter; 0 = absence of shelter).

### 2.5. Measurement of Tree Canopy Surrounding Bus Stops

Another exposure variable of interest was the percentage of tree canopy surrounding bus stops. We developed this variable from four elements: shapefiles of bus stops, streets, and building footprints downloaded online from open data portals of the state of Texas and city of Austin [42,43,44], and aerial imagery acquired in November 2018 by the National Agriculture Imagery Program (NAIP) of the US Department of Agriculture [45]. Previous studies have used NAIP imagery to classify vegetation and other land cover because of its capture during the agricultural growing season (i.e., “leaf-on” season), high spatial resolution (i.e., typical ground sample distance of one meter), and four spectral bands (i.e., red, green, blue, and near infrared) for image classification [30,46,47,48]. To develop the variable for tree canopy, we first imported all elements into a geographic information system (ArcGIS 10.8, ESRI, Redlands, CA, USA). From a mosaicked NAIP aerial image covering the spatial extent of the Capital Metro bus system, we performed iso-cluster unsupervised classification to categorize four spectral bands into 100 unique information classes [30] based on reflectance values of image pixels. Next, we manually reclassified pixels into two unique classes: Tree Canopy and Not Tree Canopy.

We conducted an accuracy assessment of image classification by comparing sample points, generated using simple random selection, on both classified data and reference data (i.e., NAIP aerial imagery). Aiming for an accuracy of greater than 85% allocation, the standard accuracy threshold [49], we calculated the need for 196 sample points from an equation based on binomial probability theory [50]. The accuracy assessment revealed that classification was above the 85% accuracy threshold, on average and for each of the two classes. Overall accuracy (i.e., the number of sample points classified correctly divided by the total number of sample points) was 93.4%. Producer’s accuracy (i.e., omission errors where pixels were excluded from a class when in reality belonging to that class) was 95.0% for Tree Canopy and 92.6% for Not Tree Canopy. User’s accuracy (i.e., commission errors where pixels were included in a class when in reality not belonging to that class) was 85.1% for Tree Canopy and 97.7% for Not Tree Canopy.

**Figure 2:**
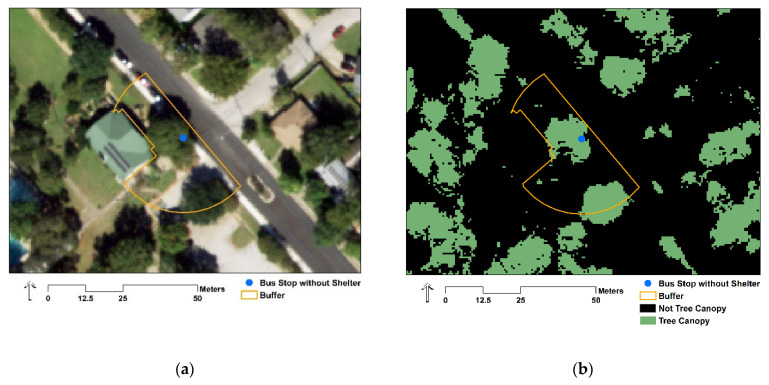
(**a**) Buffer to calculate the percentage of tree canopy surrounding a bus stop (aerial image); (**b**) buffer to calculate the percentage of tree canopy surrounding a bus stop (Not Tree Canopy and Tree Canopy classes).

To measure only tree canopy surrounding bus stops, we used a Euclidean buffer of 25 m—roughly equal to a 20-s walk [51]—around each stop. We believed 25 m was a reasonable distance from a bus stop to seek tree shade without missing the next bus. We then clipped each buffer to remove portions on the opposite side of the street from bus stops to focus on tree canopy that individuals could readily access. We also clipped each buffer to remove building footprints and to improve focus on publicly accessible outdoor space (Figure 2). Lastly, we performed zonal statistics to calculate the percentage of pixels within each buffer classified as Tree Canopy.

### 2.6. Measurement of Sociodemographic Characteristics

To understand the distribution of shelters and tree canopy surrounding bus stops in relation to the potential users nearby, we measured the percentage of different population groups within the census tract of each bus stop: (a) non-Hispanic white, (b) Hispanic all races, (c) non-Hispanic black, (d) non-Hispanic Asian, (e) individuals below federal poverty level, and (f) workers who take the bus to work. We focused on these population groups because heat exposure has been found to differ based on race, ethnicity, and class of the community [26], and most public transit users are subjected to weather conditions when reaching their stop or station [8]. We also measured median age, in years, within the census tract of each bus stop, because older adults are at a higher risk of heat-related illness than younger adults [52], and therefore, can particularly benefit from adaptation strategies for extreme heat. All sociodemographic data were five-year estimates from the 2014–2018 American Community Survey [53,54,55,56]. Among Austin residents in 2019, 48.3% identified as non-Hispanic white, 34.3% identified as Hispanic, 7.8% identified as non-Hispanic black, 7.3% identified as non-Hispanic Asian, 14.5% were considered in poverty, 2.2% take the bus to work, and 8.9% were ages 65 years and over [31,57].

### 2.7. Statistical Analyses

We first calculated summary statistics (i.e., mean, standard deviation, median, interquartile range, minimum, and maximum) for each study variable. We derived summary statistics for day-level variables (i.e., boardings, service frequency, Tmax, Hi-T Day, and precipitation) by averaging within each bus stop and then averaging across bus stops. We then used multilevel negative binomial regression models to test whether shelters and tree canopy surrounding bus stops (a) impacted the effect of temperatures on bus ridership and (b) were equitably distributed. Multilevel negative binomial modeling properly accounted for clustering of days of observation within each bus stop (via a random intercept for each stop), and the dependent variable consisted solely of non-negative integers. We treated total ridership per day as the dependent variable, and included an exposure term for the number of bus arrivals per day at each stop to effectively model ridership per bus arrival.

To assess whether shelters and tree canopy surrounding bus stops impacted the effect of temperatures on boardings per bus, we employed two sets of models—one for weekdays and another for weekends—because of differences in bus transit use on Monday through Friday versus Saturday and Sunday [37]. Specifically, each set included a series of five models with different exposure variables of interest: (a) daily maximum air temperature, (b) interaction between daily maximum air temperature and presence of shelter, (c) interaction between daily maximum air temperature and percentage of tree canopy surrounding bus stop, (d) interaction between high-temperature day and presence of shelter, and (e) interaction between high-temperature day and percentage of tree canopy surrounding the bus stop. To aid interpretability, we reported model results as incidence-rate ratios (i.e., exponentiated raw regression coefficients).

We adjusted final models for aforementioned sociodemographic characteristics (i.e., non-Hispanic white, Hispanic all races, non-Hispanic black, non-Hispanic Asian, individuals below poverty level, workers who take the bus to work, and median age) and five other potential confounders. We adjusted for daily precipitation (cm), which has been found to be negatively associated with ridership [10,13,14,15,16,19,20]. Precipitation data originated from Camp Mabry weather station in the same daily summaries dataset as air temperature data [35]. We also included the service frequency, density of bus stops, and access to bus transit as three potential confounders, because each has been shown to exhibit significant associations with ridership [20,58]. For service frequency, Capital Metro provided data on the total number of times a bus arrived at a bus stop between 13:00 and 18:00 each study day. For bus stop density and access to bus transit, we imported shapefiles of bus stops and bus routes into GIS to calculate the number of bus stops and bus routes, respectively, within an 800 m service area of each bus stop [42]. In addition, we used five-year estimates from the 2014–2018 American Community Survey to measure the total population within the census tract of each bus stop, which may influence bus ridership [53].

Finally, we estimated two models for the presence of shelter and percentage of tree canopy surrounding bus stops as functions of percentages of non-Hispanic white, Hispanic all races, non-Hispanic black, non-Hispanic Asian, individuals below poverty level per census tract, and workers who take the bus to work, along with median age. We adjusted models for the service frequency, density of bus stops, access to bus transit, and total population. For the bus shelter model, we used logistic regression and presented results as odds ratios (i.e., exponentiated regression coefficients). We used linear regression for the tree canopy model. All statistical analyses were completed in Stata 15.1 (StataCorp, College Station, TX, USA). Data presented in this study and associated metadata are openly available in FigShare at https://doi.org/10.6084/m9.figshare.13322597.v2 [59].

## 3. Results

### 3.1. Summary Statistics

On study days (n = 183) between 13:00 and 18:00, average boardings decreased 37.9% from weekdays to weekends, while service frequency was relatively consistent (Table 1). Daily maximum air temperature averaged 33.73 °C (standard deviation = 0.62 °C) per stop, with 44% of days considered high-temperature days. In terms of absolute day-level values (data not shown in Table 1), the lowest and highest values of daily maximum air temperature were 17.78 °C and 40.56 °C, respectively, and only three study days (1.6%) experienced rain. Among bus stops (n = 2271), 29% had shelters. Tree canopy surrounding bus stops averaged 14.29% (standard deviation = 14.46%), and ranged 0–74%. Bus stops were located in census tracts with the majority of population identifying as white (mean = 47.99%; standard deviation = 21.43%) or Hispanic (mean = 35.585%; standard deviation = 20.51%), and about one in five below poverty level (mean = 18.07%; standard deviation = 12.28%). In tracts with bus stops, the percentage of bus commuters was relatively low, on average (mean = 4.84%; standard deviation = 5.11%).

### 3.2. Relations between Bus Stop Shelters, Tree Canopy, and Ridership in Warm Season Temperatures

In assessing whether shelters and tree canopy surrounding bus stops impacted the effect of temperatures on boardings per bus, we described herein the five-model set for weekdays (Table 2), since we found near identical results as the model set for weekends (Table A1 in Appendix A). Each one-degree Celsius increase in daily maximum air temperature was associated with a 0.2% decrease in boardings per bus when there was no shelter at a bus stop, and a greater decrease (0.4%) in boardings per bus when there was a shelter (*p* < 0.001). When no tree canopy surrounded a bus stop, each one-degree increase in daily maximum air temperature was associated with a 0.4% decrease in boardings per bus (*p* < 0.001), with each one-percent increase in tree canopy associated with a lesser decrease (< 0.4%) in boardings per bus (*p* < 0.001). On high-temperature days, both bus stops with shelters and without shelters exhibited statistically insignificant associations with boardings per bus compared to all other days (*p* > 0.05). On high-temperature days, zero tree canopy surrounding a bus stop was associated with a 1.7% decrease in boardings per bus compared to all other days (*p* < 0.001), with each one-percent increase in tree canopy associated with a lesser decrease (1.6%) in boardings per bus (*p* < 0.001).

### 3.3. Relations between Sociodemographic Characteristics, Bus Stop Shelters, and Tree Canopy

In assessing whether bus stop shelters were equitably distributed among different populations (Table 3), each one-year increase in the median age of individuals within the census tract of a bus stop was associated with a 4.2% decrease in likelihood of a shelter at a bus stop (*p* < 0.05). Race, ethnicity, poverty level, and bus commuting did not exhibit significant associations with the presence of a shelter (*p* > 0.05). In assessing whether tree canopy surrounding bus stops was equitably distributed among different populations (Table 4), each one percentage point increase in bus commuters within the census tract of a bus stop was associated with a 0.17 percentage point decrease in tree canopy surrounding a bus stop (*p* < 0.05). Race, ethnicity, poverty level, and median age did not exhibit significant associations with the percentage of tree canopy surrounding a bus stop (*p* > 0.05).

## 4. Discussion

In exploring the temperature–ridership relationship, our finding that warm season temperatures exhibited a significant, yet modest, negative association with bus ridership corroborated results from previous studies [11,13,19,20]. This finding suggests the need to adapt bus transit systems to protect against ridership losses induced by temperature increases during the warm season. Yet, the two climate change adaptation strategies investigated in this study—bus stop shelters and trees—exhibited insignificant or modest associations with ridership on high-temperature days, which may be attributed to the transit dependency of transit users. In Austin, those who take public transportation to work have the lowest median earnings of all workers (e.g., 41.6% lower than those who drove automobiles alone to work) [60], and more than a quarter of these individuals have no vehicle available [61]. Transit-dependent individuals have no choice but to use bus stops, regardless of whether shelters or nearby trees are present to provide respite from adverse heat conditions.

Our finding of a greater negative association between warm season temperatures and boardings for bus stops with shelters compared to stops without shelters may be related to microclimatic differences of locations with shelters versus those without shelters. Since Capital Metro determines whether a bus stop qualifies for a shelter based on the generated number of daily boardings and nearness to high-activity areas (e.g., apartments and employment centers) [41], bus stops with shelters may be located in denser, more urban areas than stops without shelters. As such, locations with shelters may experience higher temperatures than locations without shelters because of heat islands driven by high amounts of impervious materials and waste heat emissions, lack of trees, and urban geometry [25]. These potentially higher temperatures at locations with shelters would not have been captured by the daily air temperature measurement from Camp Mabry weather station, and may have led to reduced ridership because of thermal discomfort experienced at these locations. Conversely, our finding that tree canopy surrounding bus stops moderated the effect of warm season temperatures on ridership may be attributed to bus stops with trees being in areas characterized by lower temperatures, in part because of trees’ ability to decrease ambient temperatures through shading and evapotranspiration [25].

Lastly, our findings that race, ethnicity, and poverty level of populations within census tracts of bus stops did not exhibit significant associations with locations of shelters and tree canopy surrounding bus stops may provide evidence for even spatial coverage of these bus stop amenities among these populations in Austin. The significant negative association between the median age of individuals within the tract of a bus stop and presence of a shelter may be related to Capital Metro service guidelines and standards for shelter placement based on ridership levels [41]; Capital Metro may not be installing shelters in tracts with higher median age, since older adults have been shown to ride public transit less than other populations [8]. The modest negative association between the percentages of bus commuters and tree canopy may provide evidence of an inequity, with tracts with more bus commuters having less access to trees.

Based on study findings, cities located in humid subtropical climates should consider extreme heat as a threat to public transportation use and tree planting around bus stops as a climate change adaptation strategy to reduce ridership losses. Although we did not find bus stop shelters to mitigate ridership losses during warm season temperatures, we believe shelters remain a viable adaptation strategy because these roofed structures provide protection from direct solar radiation and precipitation [20]. Pairing trees and shelters at bus stops can improve thermal comfort of bus transit users, lower their risk of heat-related illness on high-temperature days, and potentially mitigate ridership losses. Along with cooling, trees at bus stops can provide a host of other environmental and health benefits. Street trees, for instance, have been found to diminish noise pollution, capture airborne pollutants, sequester carbon dioxide, and reduce stormwater runoff [62]. Moreover, researchers have found transit users to underestimate the wait time for the bus when stops were surrounded by dense, mature tree cover [63].

This study has limitations that can be addressed in future work. First, we utilized air temperature data from a single weather station for all bus stops in our study, when in reality, air temperatures may differ substantially across a city [64]. Weather stations installed at bus stops or wearable sensors worn by bus transit users, such as Thermochron iButtons [65], can better capture experienced microclimatic conditions. To determine how heat stress—consisting of weather parameters of air temperature, relative humidity, wind speed, and solar radiation and human-related parameters of activity level and clothing [66]—relates to adaptation strategies and bus ridership, researchers can utilize one of 162 human thermal climate indices, such as Physiological Equivalent Temperature and Universal Thermal Climate Index [67]. Second, we did not have data on bus transit users that could have confounded relations between temperature and ridership. This study would have benefitted from knowing whether individuals engaged in active transportation (e.g., walking or biking) to reach the bus stop and the length of time an individual had been outdoors subjected to ambient conditions, since both metabolic and environmental heat sources impact an individual’s level of heat stress [68]. Engaging in heat-adaptive behaviors, such as staying hydrated and wearing a hat [69], may have also confounded the temperature–ridership relationship. Furthermore, if individuals perceived temperatures to be uncomfortable, they may have decided in advance to not use bus transit, regardless of whether a shelter or tree is at the bus stop. Future studies can administer surveys to Austin residents to collect these individual-level data. As a third limitation, the variable for the percentage of tree canopy surrounding bus stops may lack validity because it (a) was developed from aerial imagery and building footprint data that were temporally mismatched with other study data; (b) assumed each percent change in tree canopy would be associated with ridership when, in reality, an individual may only require enough shade for themselves; and (c) assumed tree canopy was accessible to transit users and not on private property or behind a wall or fence. Lastly, results may be biased because of omitted variables that have been found to be associated with bus ridership, such as parks and commercial properties [58]. Subsequent studies can use the analytic audit tool or other environmental audit instruments to measure built environment features of street segments surrounding transit stops or stations [70].

## 5. Conclusions

We provided evidence for warm season temperatures in a humid subtropical climate as a barrier to bus transit use, and for tree planting around bus stops as a potentially more effective strategy than bus stop shelters in mitigating ridership losses during high temperatures. Our overall findings that shelters and tree canopy exhibited insignificant or modest associations, respectively, with ridership during high temperatures may be related to individuals’ dependence on bus transit. Criteria for shelter placement by the local transit authority may have led to the placement of shelters in areas characterized by urban heat islands. We also found shelters and tree canopy surrounding bus stops were, in general, equitably distributed among different populations. With warming from climate change and urban development projected to continue, local governments should consider how to protect from bus ridership losses and unhealthy exposure to extreme heat. Tree planting is a reasonable climate change adaptation strategy at bus stops for its multiple ecosystem services, and can be coupled with shelters for protection from multiple weather conditions. Ultimately, trees mitigate climate change and, when planted around bus stops, may make for climate-resilient public transportation that promotes mode shift away from the automobile.

## Figures and Tables

**Table 1:** Summary statistics of study variables.

	Mean	Std. Dev.	Median	IQR	Min.	Max.
**Day-Level Variables ^1^**						
Boardings Weekday (#)	14.5	40.92	3	10	0	967
Boardings Weekend (#)	9	23.97	2	8	0	651
Service Freq. Weekday (#)	18.57	14.65	19	10	1	138
Service Freq. Weekend (#)	18.08	11.84	20	10	1	135
Tmax (°C)	33.73	0.62	33.78	0.03	27.87	37.85
Hi-T Day (1 = Hi-T Day)	0.44					
Precipitation (cm)	0.3	0.04	0.3	0	0.03	0.61
**Stop-Level Variables**						
Shelter (1 = shelter)	0.29					
Tree (% canopy)	14.29	14.46	10	21	0	74
Stop Density (#)	13.38	7.59	12	10	1	38
Transit Access (#)	6.3	6.51	5	6	1	42
White (%)	47.99	21.43	50.69	34.4	5.38	91.79
Hispanic (%)	35.58	20.51	31.4	34.61	4.69	86.35
Black (%)	8.81	8.68	6.22	7.76	0	39.86
Asian (%)	5.03	5.66	2.82	4.68	0	37.5
Impoverished (%)	18.07	12.28	15.62	15.58	0.69	87.1
Bus Commuters (%)	4.84	5.11	3.76	4.1	0	80
Median Age (years)	33.09	4.37	33.2	5.4	19.8	50.8
Total Population (#)	5366.97	2207.78	4949	2818	0	13,422

^1^ Statistics were derived by averaging within each bus stop and then averaging across bus stops.

**Table 2:** Model output for relations between temperature, bus stop shelters, tree canopy, ridership, and weekday.

	(1) Tmax	(2) Tmax X Shelter	(3) Tmax X Tree	(4) Hi-T Day X Shelter	(5) Hi-T Day X Tree
**Day-Level Variables**					
Tmax (°C)	0.997 *** (−14.80)	0.998 *** (−7.67)	0.996 *** (−15.28)		
Hi-T Day (1 = Hi-T Day)				0.997 (−1.19)	0.983 *** (−6.07)
Precipitation (cm)	0.983 *** (−15.33)	0.983 *** (−15.34)	0.983 *** (−15.33)	0.987 *** (−12.33)	0.987 *** (−12.33)
**Stop-Level Variables**					
Shelter (1 = shelter)		4.391 *** (25.36)		4.138 *** (25.05)	
Tree (% canopy)			0.978 *** (−11.20)		0.980 *** (−10.10)
Stop Density (#)	1.008 (1.56)	1.000 (0.08)	1.006 (1.14)	1.000 (0.09)	1.006 (1.14)
Transit Access (#)	1.053 *** (8.48)	1.045 *** (8.07)	1.049 *** (8.05)	1.045 *** (8.22)	1.049 *** (8.08)
White (%)	1.034 (1.89)	1.022 (1.34)	1.030 (1.70)	1.022 (1.33)	1.030 (1.69)
Hispanic (%)	1.050 ** (2.85)	1.035 * (2.21)	1.045 * (2.56)	1.035 * (2.19)	1.045 * (2.56)
Black (%)	1.029 (1.57)	1.015 (0.93)	1.024 (1.30)	1.015 (0.92)	1.024 (1.30)
Asian (%)	1.067 *** (3.46)	1.052 ** (3.02)	1.062 ** (3.24)	1.052 ** (3.00)	1.062 ** (3.24)
Impoverished (%)	1.003 (0.79)	1.000 (0.10)	1.004 (1.12)	1.000 (0.10)	1.004 (1.12)
Median Age (years)	0.982 (−1.77)	0.996 (−0.50)	0.985 (−1.51)	0.996 (−0.50)	0.985 (−1.51)
Total Population (#)	1.000 (−0.56)	1.000 (0.42)	1.000 (−0.81)	1.000 (0.42)	1.000 (−0.81)
**Interaction Terms**					
Tmax X Shelter		0.998 *** (−4.47)			
Tmax X Tree			1.000 *** (6.26)		
Hi-T Day X Shelter				0.993 (−1.67)	
Hi-T Day X Tree					1.001 *** (5.51)
Lnalpha	0.102 *** (−335.62)	0.102 *** (−335.60)	0.102 *** (−335.67)	0.102 *** (−335.45)	0.102 *** (−335.50)
Constant	6.228 *** (32.70)	4.142 *** (32.55)	5.776 *** (32.7 1)	4.142 *** (32.55)	5.775 *** (32.71)
*N*	290,692	290,692	290,692	290,692	290,692

Exponentiated coefficients; *t* statistics in parentheses, * *p* < 0.05, ** *p* < 0.01, *** *p* < 0.001. Dependent variable: boardings per bus between 13:00 and 18:00 (#).

**Table 3:** Model output for relations between sociodemographic characteristics and bus stop shelters.

	(1) Shelter
**Variables of Interest**	
White (%)	1.054 (1.59)
Hispanic (%)	1.063 (1.91)
Black (%)	1.058 (1.70)
Asian (%)	1.060 (1.69)
Impoverished (%)	1.005 (0.94)
Bus Commuters (%)	1.017 (1.67)
Median Age (years)	0.958 * (−2.42)
**Potential Confounders**	
Service Frequency (%)	1.030 *** (7.18)
Stop Density (#)	1.031 *** (3.49)
Transit Access (#)	0.996 (−0.37)
Total Population (#)	1.000 (−1.47)
*N*	2271

Exponentiated coefficients; * *p* < 0.05, ** *p* < 0.01, *** *p* < 0.001. Dependent variable: presence of shelter (1 = presence; 0 = absence).

**Table 4:** Model output for relations between sociodemographic characteristics and tree canopy surrounding bus stops.

	(1) Tree
**Variables of Interest**	
White (%)	−0.185 (−0.93)
Hispanic (%)	−0.259 (−1.35)
Black (%)	−0.257 (−1.28)
Asian (%)	−0.222 (−1.06)
Impoverished (%)	0.0719 (1.96)
Bus Commuters (%)	−0.166 * (−2.50)
Median Age (years)	0.0822 (0.77)
**Potential Confounders**	
Service Frequency (%)	−0.113 *** (−4.46)
Stop Density (#)	−0.110 * (−1.99)
Transit Access (#)	−0.0848 (−1.21)
Total Population (#)	−0.000290 (−1.79)
Constant	38.16 * (2.06)
*N*	2271

Coefficients; * *p* < 0.05, ** *p* < 0.01, *** *p* < 0.001. Dependent variable: tree canopy surrounding bus stops (%).

## Data Availability

Data presented in this study and associated metadata are openly available in FigShare at https://doi.org/10.6084/m9.figshare.13322597.v2 [59].

## Appendix A

**Table A1:** Model output for relations between temperature, bus stop shelters, tree canopy, ridership, and weekend.

	(1) Tmax	(2) Tmax X Shelter	(3) Tmax X Tree	(4) Hi-T Day X Shelter	(5) Hi-T Day X Tree
**Day-Level Variables**					
Tmax (°C)	0.996 *** (−8.75)	0.997 *** (−4.72)	0.995 *** (−8.87)		
Hi-T Day (1 = Hi-T Day)				0.998 (−0.35)	0.989 * (−1.98)
Precipitation (cm)	0.969 *** (−18.62)	0.969 *** (−18.62)	0.969 *** (−18.62)	0.974 *** (−16.60)	0.974 *** (−16.60)
**Stop-Level Variables**					
Shelter (1 = shelter)		3.989 *** (21.17)		3.724 *** (22.07)	
Tree (% canopy)			0.978 *** (−9.66)		0.981 *** (−9.23)
Stop Density (#)	1.009 (1.64)	1.003 (0.62)	1.006 (1.17)	1.003 (0.63)	1.006 (1.17)
Transit Access (#)	1.034 *** (5.30)	1.027 *** (4.66)	1.032 *** (5.05)	1.027 *** (4.66)	1.032 *** (5.05)
White (%)	1.038 (1.84)	1.025 (1.35)	1.033 (1.66)	1.025 (1.34)	1.033 (1.66)
Hispanic (%)	1.051 * (2.53)	1.035 (1.95)	1.045 * (2.30)	1.035 (1.95)	1.045 * (2.30)
Black (%)	1.027 (1.31)	1.013 (0.73)	1.022 (1.09)	1.013 (0.73)	1.022 (1.08)
Asian (%)	1.058 ** (2.66)	1.044 * (2.28)	1.054 * (2.51)	1.044 * (2.27)	1.054 * (2.50)
Impoverished (%)	1.001 (0.25)	0.999 (−0.31)	1.002 (0.47)	0.999 (−0.31)	1.002 (0.47)
Median Age (years)	0.988 (−1.09)	1.000 (−0.02)	0.990 (−0.95)	1.000 (−0.02)	0.990 (−0.95)
Total Population (#)	1.000 (0.02)	1.000 (0.72)	1.000 (−0.29)	1.000 (0.72)	1.000 (−0.29)
**Interaction Terms**					
Tmax X Shelter		0.998 ** (−2.64)			
Tmax X Tree			1.000 *** (3.35)		
Hi-T Day X Shelter				0.993 (−0.77)	
Hi-T Day X Tree					1.001 (1.61)
Lnalpha	0.162 *** (−159.71)	0.162 *** (−159.70)	0.162 *** (−159.73)	0.162 *** (−159.62)	0.162 *** (−159.64)
Constant	6.437 *** (30.81)	4.450 *** (30.57)	5.978 *** (30.78)	4.449 *** (30.57)	5.976 *** (30.78)
*N*	102,915	102,915	102,915	102,915	102,915

Exponentiated coefficients; *t* statistics in parentheses, * *p* < 0.05, ** *p* < 0.01, *** *p* < 0.001. Dependent variable: boardings per bus between 13:00 and 18:00 (#).
